# A Novel Reporter Mouse Uncovers Endogenous Brn3b Expression

**DOI:** 10.3390/ijms20122903

**Published:** 2019-06-14

**Authors:** Adam M. Miltner, Yesica Mercado-Ayon, Simranjeet K. Cheema, Pengfei Zhang, Robert J. Zawadzki, Anna La Torre

**Affiliations:** 1 Department of Cell Biology and Human Anatomy, University of California-Davis, Davis, CA 95616, USA; ammiltner@ucdavis.edu (A.M.M.); mercadoayon@ucdavis.edu (Y.M.-A.); simcheema@ucdavis.edu (S.K.C.); pfzhang@ucdavis.edu (P.Z.); 2 UC Davis EyePod Small Animal Ocular Imaging Laboratory, University of California-Davis, Davis, CA 95616, USA; rjzawadzki@ucdavis.edu; 3 Department of Ophthalmology and Vision Science, University of California-Davis, Sacramento, CA 95817, USA

**Keywords:** retinal ganglion cells (RGCs), CRISPR-Cas9, retinal development, optic stalk, retinal imaging, scanning laser ophthalmoscopy (SLO)

## Abstract

Brn3b (*Pou4f2*) is a class-4 POU domain transcription factor known to play central roles in the development of different neuronal populations of the Central Nervous System, including retinal ganglion cells (RGCs), the neurons that connect the retina with the visual centers of the brain. Here, we have used CRISPR-based genetic engineering to generate a Brn3b-mCherry reporter mouse without altering the endogenous expression of Brn3b. In our mouse line, mCherry faithfully recapitulates normal Brn3b expression in the retina, the optic tracts, the midbrain tectum, and the trigeminal ganglia. The high sensitivity of mCherry also revealed novel expression of Brn3b in the neuroectodermal cells of the optic stalk during early stages of eye development. Importantly, the fluorescent intensity of Brn3b-mCherry in our reporter mice allows for noninvasive live imaging of RGCs using Scanning Laser Ophthalmoscopy (SLO), providing a novel tool for longitudinal monitoring of RGCs.

## 1. Introduction

Retinal ganglion cells (RGCs) are the sole output neurons of the retina. Located along the inner surface of the retina, RGCs receive and integrate signals from the retina circuitry and send long axons that converge at the center of the eye to form the optic nerve and convey visual information to several areas of the brain [1,2]. There are approximately 40 RGC subtypes classified by single-cell RNA sequencing clustering algorithms [3], and RGCs have also been categorized into numerous subtypes based on their morphological and functional properties [4]. Different RGC populations participate in distinct circuits, but the vast majority of optic nerve axons terminate in the lateral geniculate nucleus of the thalamus or the superior colliculus in the tectum of the midbrain [5].

Progressive damage to the optic nerves and RGC degeneration is the final common pathway that leads to vision loss in glaucoma, one of the leading causes of visual impairment and blindness worldwide [6]. Although research in the field of glaucomatous degenerations is extensive, the pathophysiological mechanisms underlying these diseases are not completely understood. Rodent models of RGC degeneration have been vital to our continued efforts to understand the progression of glaucoma and to devise novel therapeutic interventions. Several invasive methods [7,8,9,10,11,12,13,14], naturally occurring models [15,16], and genetic manipulations [17,18] have been utilized as a proxy to glaucomatous disease. However, all these methods rely on in vivo optical coherence tomography scans (OCT) imaging [19] or post-mortem histological procedures to assess the degree of RGC degeneration. In general, OCT measurements of the ganglion cell complex involve measuring the thickness of three layers together: the inner plexiform layer (IPL) that contains the dendritic arbors of the RGCs and the axons of the cells of the inner nuclear layer of the retina, the ganglion cell layer (GCL) that contains the somas of the RGCs and the displaced amacrine cells, and the retinal nerve fiber layer (RNFL) containing the RGC axonal processes and astrocytes [20,21]. While great progress in segmentation algorithms has been achieved and measurements of the GCL alone are possible, albeit time-consuming, and novel imaging technologies are being developed, the current standard tools are not ideal to follow RGCs over time [22]. Similarly, several RGC reporter mice are available including Thy-XFP, Isl2-GFP, DRD4-GFP, Hoxd10-GFP and many others but these reporters are either not specific or label only a small subset of RGCs [23,24,25,26,27,28,29].

Previous reports have identified Brn3b (Pou4f2), a POU-4 transcription factor, as a master regulator of RGC development and neural cell type diversity [30,31,32]. The basic helix-loop-helix (bHLH) transcription factor Atoh7/Math5 is expressed in RGC precursors and controls the expression of Brn3b and another transcription factor, Isl1, which in a combinatorial manner are required for the initiation of the RGC program [33,34]. Consequently, deletion of Brn3b results in the loss of 70% of all RGCs, with remaining RGCs exhibiting axonal guidance defects or delays [35,36,37,38,39,40]. Conversely, overexpression of Brn3b and Isl1 together is sufficient to promote RGC fates [41]. Brn3b expression has also been observed in the trigeminal nerve ganglia and other cranial nerve nuclei in a very characteristic and dynamic spatiotemporal pattern [42,43]. 

Here, we have developed a murine fluorescent reporter using CRISPR-Cas9 to introduce monomeric Cherry fluorescent protein (mCherry) after the coding sequence of Brn3b, without modifying its normal expression. In Brn3b-mCherry mice, mCherry closely recapitulates endogenous Brn3b expression and, as a result, labels a large proportion of RGCs. In the retina, mCherry expression begins around embryonic day 11.5 (E11.5), coinciding with the onset of RGC genesis, and persists throughout development and in the adult retina. Interestingly, our approach also revealed novel Brn3b expression in the optic stalk during early development. Additionally, the high detectability of mCherry allows for live imaging of RGCs using fluorescence detection by Scanning Laser Ophthalmoscopy (SLO), providing a powerful tool to monitor RGCs over time.

## 2. Results and Discussion

### 2.1. Generation of CRISPR–Engineered Brn3b-mCherry Reporter Mouse Line

**Figure 1:**
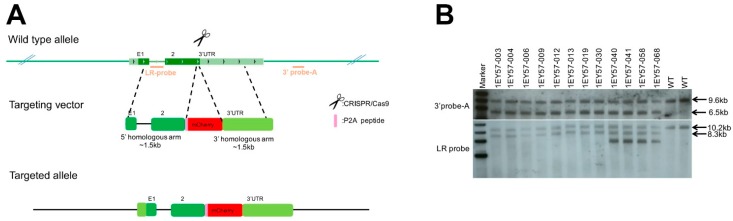
Design and validation of CRISPR/Cas9 strategy. (**A**) Schematic of the mouse Brn3b locus. The dark green boxes correspond to the two exons of Brn3b (E1/2). Two probes named LR-probe and 3′ probe-A (orange lines) were used to identify correctly targeted animals. (**B**) Southern blot of Brn3b-mCherry mice showing correct insertion of the mCherry targeting vector into the Brn3b locus. Further details on Southern blot design strategy design is detailed in Figure S1.

We aimed to generate a Brn3b reporter mouse line without interfering with Brn3b endogenous expression. To that goal, we designed a CRISPR/Cas9 (Clustered Regularly Interspaced Short Palindromic Repeats/CRISPR-associated protein 9) strategy to insert 2A self-cleaving peptide (P2A) and monomeric Cherry fluorescent protein (mCherry) sequences immediately downstream of the coding sequence of the Brn3b gene (Pou4f2, transcript NM_138944, Figure 1A). The gene-targeting was completed by Biocytogen (Worcester, MA, USA). Briefly, single guide RNAs (sgRNAs) were designed using the CRISPR Finder design tool www.sanger.ac.uk [44]. One sgRNA (GGAGAAGGGTCCCTAAATGC) was selected, cloned into pT7-sgRNA by Gibson assembly, confirmed by DNA sequencing, and transcribed in vitro. Similarly, the targeting vector was constructed as shown in Figure 1A. and validated by DNA sequencing. 286 zygotes were microinjected and transferred into pseudopregnant females, from which 21 pups were born. With this strategy, a cassette containing a P2A-mCherry sequence flanked by homology arms to exon 2 of Brn3b (5′ homology) and to the 3′ UTR of the Brn3b coding sequence (3′ homology) were inserted by homologous recombination into the Brn3b locus. Therefore, the resulting animals express Brn3b and mCherry from the endogenous Brn3b promoter and regulatory elements. The founders were bred, the F1 animals were genotyped, and the positive animals were further confirmed by Southern blot using two different probes (Figure 1B) as detailed in Figure S1.

Other Brn3b reporter animals have been previously reported, including a GFP knock-in reporter [45] as well as numerous strategies for labeling Brn3b+ cells using Cre or Dre-driven recombination combined with alkaline phosphatase or lacZ to visualize the recombined cells [46,47,48,49]. Each of these models is useful for studying RGC and retinal biology, however in all these lines at least one copy of Brn3b is disrupted. Furthermore, Brn3b is present in the germline or ubiquitously expressed in the early stages of development leading to recombination in the whole animal in some Brn3b-Cre knock-in models [49]. 

Similarly, other fluorescent RGC reporters using different drivers have also been developed. For example, several Thy1 reporters have been generated and are widely used. However, Thy1 is not exclusively expressed in RGCs as many displaced amacrine cells as well as INL neurons also express Thy1 [23]. It is important to note that in our design we chose mCherry because it typically displays low autofluorescence background levels and high photostability [50]. Furthermore, our Brn3b-mCherry line could be easily combined with other existing RGC reporters such as the Thy1-YFP or Isl2-GFP mice [23,51], and a similar strategy has been successfully used to label RGCs derived from human Embryonic Stem Cells [52,53].

### 2.2. mCherry Labels A Large Fraction of RGCs in the Adult Retina of Brn3b-mCherry Mice

**Figure 2:**
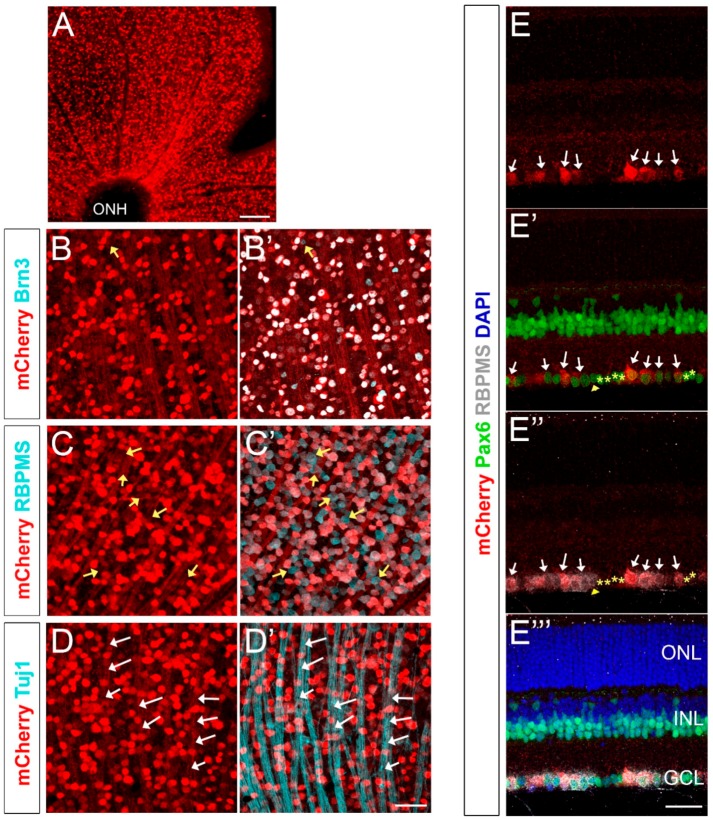
Brn3b-mCherry expression in the adult retina. (**A**) Flat-mounted retina labeled with anti-mCherry antibody. (**B**,**B’**) mCherry (red) and Brn3 (teal) colocalization. Yellow arrow indicates a Brn3+ mCherry- cell. (**C**,**C’**) mCherry (red) and RBPMS (teal) colocalization. Yellow arrows indicate RBPMS+ mCherry- cell bodies. (**D**,**D’**) mCherry (red) and Tuj1 (teal) colocalization. White arrows indicate Tuj1+ mCherry+ axons. (**E**–**E’’’**) Cross-section of an adult retina labeled with mCherry (red), RBPMS (gray), DAPI (blue), and Pax6 (green). All mCherry+ cells (white arrows) are RBPMS+. Amacrine cells are labeled with yellow stars and are mCherry- (Pax6+ RBPMS− mCherry− cells). Yellow arrowhead corresponds to an mCherry− RGC (RBPMS+ Pax6− mCherry− cell). ONH: Optic Nerve Head. ONL: Outer Nuclear Layer. INL: Inner Nuclear Layer. GCL: Ganglion Cell Layer. Scale bars: 300 microns in A, 50 microns in B–E’’’.

To analyze mCherry expression in the adult retina, 8–12-week old Brn3b-mCherry mice (*n* = 4 from two different generations) were euthanized, and their retinas were dissected, flat-mounted, fixed, and immunolabeled with mCherry and Brn3, as well as with other known RGC markers such as RBPMS and Tuj1 (Figure 2). mCherry+ RGC somas range from 8.25–29.67 μm, with an average diameter of 15.1 +/− 3.6 μm consistent with previous reports [29], and we observed an average of 2297 +/− 411 mCherry+ cells/mm^2^ of retina (mean +/− SD, Table S1). A widely-used pan-Brn3 antibody that detects Brn3a, Brn3b and Brn3c (C-20, Santa Cruz Biotechnology) labels most RGCs, but some subpopulations, including some intrinsically-photosensitive RGCs (ipRGCs), are Brn3− [54,55]. As expected, mCherry highly colocalizes with Brn3 (99.06 ± 0.25% colocalization, indicated as mean +/− SD) and we only found a very small subset of Brn3+ cells that were mCherry− (yellow arrow in Figure 2B’), presumably indicating a small fraction of cells that are Brn3b− but Brn3a+ or Brn3c+. As described previously, RBPMS specifically labels all RGCs [29]. Counting RBPMS+ and mCherry+ cells in our Brn3b-mCherry mouse indicated that 70.86 ± 4.33% of all RGCs are mCherry+ (data indicated as mean +/− SD, yellow arrows in Figure 2C’ show RBPMS+ mCherry− RGCs). Conversely, 100% of mCherry+ cells are RBPMS+ indicating that all fluorescently-labeled cells are RGCs (Figure 2C,C’). Since the mCherry sequence does not include a nuclear localization signal, the RGC axonal bundles (Tuj1+, white arrows in Figure 2D’) and optic nerves also exhibit detectable levels of mCherry. 

Paraffin sections of Brn3b-mCherry adult mice showed specific mCherry expression only in the GCL (Figure 2E). To verify that mCherry is only expressed in RGCs and not in displaced amacrine cells or in any other retinal cell type, we performed co-localization experiments with Pax6 (Figure 2E’) and RBPMS (2E”). Pax6 is expressed in RGCs, amacrine cells, horizontal cells, and Muller glia [56]. Therefore, Pax6+ RBPMS- cells located in the GCL are displaced amacrine cells (yellow stars in Figure 2E’,E’’) and these cells are not mCherry positive. Conversely, a considerable fraction of RBPMS+ cells exhibit mCherry expression (white arrows, Figure 2E–E’’), similarly to our flat-mount experiments. Together, these findings suggest that our Brn3b-mCherry reporter mouse specifically labels RGCs in the adult mouse retina. 

### 2.3. The Expression Pattern of mCherry Recapitulates Endogenous Brn3b during Retinal Development

**Figure 3:**
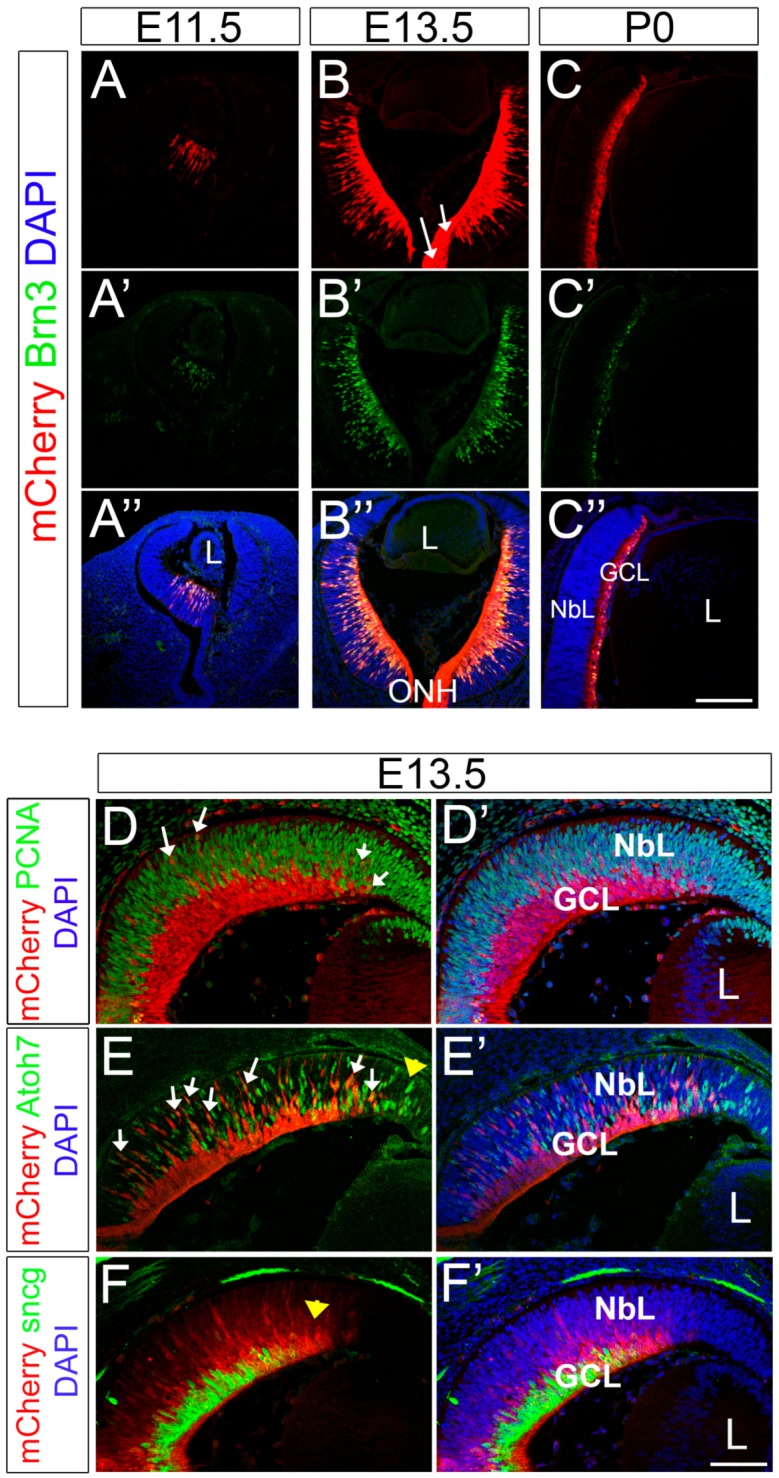
Brn3b-mCherry expression during early retinal development. (**A**–**C’’**) Co-localization experiments of mCherry and pan-Brn3 antibody at E11.5 (A-A’’), E13.5 (**B**–**B’’**) and P0 (**C–C’’**). White arrows in B indicate mCherry+ RGC axons. **D–F’**) E13.5 retina stainings. (**D**,**D’**) mCherry (red) and PCNA (green) co-localization. White arrows: PCNA+ mCherry+ cells. (**E**,**E’**) mCherry (red) and Atoh7 (green) co-localization. White arrows: mCherry+ Atoh7+ cells. Yellow arrowhead indicates the leading edge of neurogenesis. (**F**,**F’**) mCherry (red) and γ-synuclein (green) colocalization. Yellow arrowhead indicates the leading edge of neurogenesis. L: Lens. NbL: Neuroblastic Layer. GCL: Ganglion Cell layer. Scale bars: 200 microns A–C’’, 100 microns D–F’.

During normal development, retinal progenitor cells give rise to all the different cells of the retina in a stereotyped sequence with the RGCs being the first cell population generated [57,58,59]. Classic H^3^-thymidine labeling and cell lineage experiments established that in the mouse retina the onset of RGC genesis is around embryonic day 11.5 (E11.5). We detected mCherry in our Brn3b-mCherry mouse line at the onset of Brn3b expression in nascent RGCs located in the center of the retina at E11.5 and this expression progressively spreads outward during the wave of neurogenesis that occurs during retinal development (Figure 3). At all the ages analyzed, mCherry expression colocalizes with Brn3 as observed by immunohistochemistry using mCherry and Brn3 antibodies (Figure 3A–C’’). Moreover, mCherry is also observed in axonal fibers and in the optic nerve (white arrows in Figure 3B). 

To define the dynamics of Brn3b expression, we performed co-localization experiments with well-established markers for different stages in RGC development. PCNA (Proliferating Cell Nuclear Antigen) is a known marker of retinal progenitor cells that labels dividing cells during all phases of cell cycle throughout retinal histogenesis [60]. Co-immunolabeling experiments of mCherry and PCNA showed that Brn3b and PCNA are largely expressed in two separate populations. This is not surprising, as Brn3b is mostly expressed in post-mitotic RGCs. However, we found a small fraction of mCherry+ cells that co-labeled for PCNA (white arrows in Figure 3D), indicating that Brn3b can be expressed in dividing progenitors, probably during the terminal cell division. Brn3b and Isl1 have been previously detected in EdU or BrdU-labeled cells after a short chase [61,62], suggesting that these transcription factors can be expressed during S or G2, consistent with the hypothesis that fate commitment is decided prior to the terminal mitosis [61,63]. Atoh7 (Atonal homologue 7/formerly Math5) is expressed during the terminal cell cycle in a subset of progenitor cells and is known to be required for RGC genesis [33,34,63]. Accordingly, a subset of Atoh7+ cells co-expresses Brn3b [64,65]. As expected, in our mouse model, mCherry also overlaps with a small subpopulation of Atoh7+ cells (white arrows in Figure 3E). As the retina develops in a central to peripheral gradient, Atoh7 precedes Brn3b expression (yellow arrowhead in Figure 3E) consistent with a younger developmental status at the periphery. Therefore, the expression patterns observed support the current model of transcriptional relationships of Atoh7 and Brn3b. Finally, γ-Synuclein (sncg) is a commonly used marker of RGCs, and its expression has been shown to be RGC-specific in the adult retina and in purified postnatal RGC cultures [66,67,68]. All γ-Synuclein+ cells are mCherry+ at E13.5 (Figure 3F) but, in this case, mCherry expression precedes γ-Synuclein (yellow arrow in Figure 3F), suggesting that γ-Synuclein is expressed in a later maturation stage during RGC development. Altogether, our data indicates that mCherry expression in our reporter mouse faithfully recapitulates spatial and temporal Brn3b patterns in the developing mouse retina.

### 2.4. Brn3b-mCherry Reveals the Dynamic Expression of Brn3b during CNS Development

**Figure 4:**
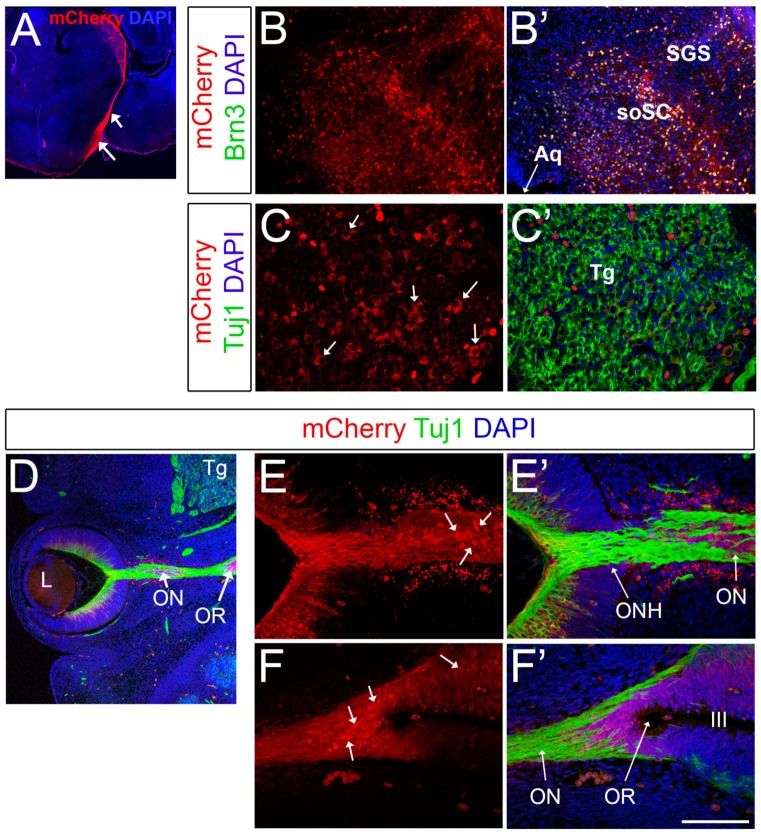
Brn3b-mCherry expression in other regions of the CNS. (**A**) Optic tracts at P0 visualized by mCherry staining (white arrows). (**B**,**B’**) Co-localization experiments with mCherry (red) and pan-Brn3 (green) antibodies at P0. (**C**,**C’**) Co-localization with mCherry (red) and Tuj1 (green) in the trigeminal ganglia at E13.5. Arrows indicate mCherry+ Tuj+ neurons. (**D**) Low magnification image of a horizontal E13.5 whole-head section. **E**–**F’**) White arrows indicate mCherry+ Tuj1- cells present in the optic stalk (**E**) and optic recess (**F**). Aq: Aqueduct. soSC: stratum opticum of the Superior Colliculus. SGS: Stratum Griseum Superficiale. Tg: Trigeminal ganglion. L: Lens. ONH: Optic Nerve Head. OR: optic recess. III: 3rd ventricle. Scale bar: 100 microns in C,C’, E,E’ and F,F’, 200 microns in B,B’, 300 microns in D, and 500 microns in A.

In addition to the expected expression in RGCs, we also validated mCherry expression in the optic nerves and optic tracts (Figure 4A). As described previously, Brn3b is also expressed in subsets of neurons of the superior colliculus, with a very dim/sparse expression in the superior layer (stratum griseum superficiale) and very prominent expression in the putative stratum opticum, where RGC axons enter the superior colliculus at a deep level relative to the pial surface [69]. In agreement with these prior findings, we detected high levels of mCherry expression in the deeper areas of the superior colliculi, and this expression greatly colocalizes with Brn3 (Figure 4B,B’). Furthermore, Brn3b is expressed in different cranial nerve nuclei in a very dynamic fashion. For example, Brn3b is present in subsets of neurons of the trigeminal ganglia (V) at early stages of development [42]. Consistently, at E13.5, mCherry is present in a salt-and-pepper manner in the trigeminal ganglia in Brn3b-mCherry mice (Figure 4C,C’).

Surprisingly, during the course of these experiments we observed mCherry expression in cells of the optic stalk (Figure 4D-F’ and Figure S2) and in neuroepithelial cells of the optic recess (Figure 4F) at E13.5. The optic stalk is the structure that connects the developing eye to the forebrain and constitutes the conduit along which the RGC axons grow; later in development, the optic stalk becomes the neuroglial sheath that surrounds the optic nerve. Interestingly, at this embryonic stage, both the optic stalk and the optic recess, which forms the boundary region between the optic vesicle, hypothalamus and telencephalon [70], are made of actively dividing neuroepithelial cells that are Pax2+ and PCNA+ (Figure S2A). This mCherry expression is intriguing since generally Brn3b is expressed in post-mitotic neurons. However, several reports indicate that Brn3b is expressed at very early stages of development, and also in the developing gonad tissues and germline cells [49,71,72]. 

In future experiments, it will be interesting to investigate the potential expression of Brn3b in the optic stalk, and to further discriminate any non-cell autonomous roles for Brn3b in optic nerve development as Brn3b-knockouts display axonal disorganization and dysfunction of RGC projections [37]. Alternatively, the presence of mCherry in the optic stalk may reflect important cellular interactions between the optic nerve axons and the neuroepithelial cells of the developing stalk such as material exchange or phagocytosis processes. Previously, transcellular degradation of axonal components by adjacent astrocytes in the optic nerve has been reported in adult mice and in frogs [73,74]. In the same direction, microglial-mediated engulfment of Brn3+ RGCs leading to the presence of Brn3-labeled fragments inside microglial cells has been observed in the embryonic retina [75]. This evidence supports previous reports indicating that a wave of RGC death at early embryonic stages regulates retinal homeostasis [75,76,77]. Since RGC axons reach the optic nerve 24–48 h after birth [78], it is possible that mechanisms of cell debris clearance, including mCherry+ axonal fragments, are taking place in the developing optic stalk. Notably, since mCherry is not as sensitive as GFP to the pH changes that occur in phagocytic vacuoles, our Brn3b-mCherry strain offers an ideal tool to investigate some of these open questions.

### 2.5. In Vivo Imaging of mCherry+ RGCs

**Figure 5:**
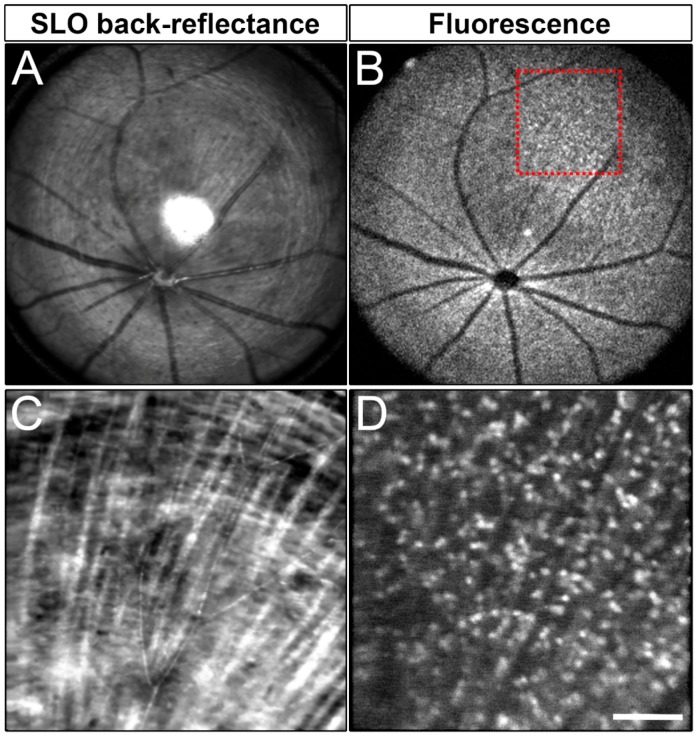
SLO imaging of a Brn3b-mCherry adult retina. (**A**) SLO back reflectance of a roughly 2mm width of an adult Brn3b-mCherry adult retina. (**B**) Fluorescent signal from the same region shown in A. (**C**) SLO back-reflectance image of the region of interest marked by a red square in B. (**D**) Fluorescent signal from the red square marked in B. Scale bars: 400 microns in A and B, and 100 microns in C and D.

Live imaging of RGCs requires relatively abundant levels of endogenous fluorescence. To address whether Brn3b-mCherry could be used as a tool to monitor RGCs in vivo, we performed noninvasive SLO using our custom multimodal mouse retinal imaging system [79]. Reflectance (Figure 5A,C) and fluorescence images (Figure 5B,D) were collected from 8–12-week-old mice (*n* = 3). Remarkably, we were able to clearly detect mCherry in the living mouse retina, indicating that the intensity of mCherry fluorescence is sufficient for in vivo imaging and longitudinal assessment of RGC status in the same animal over its lifespan. Cell counts from SLO images showed that 1139 +/− 87.88 mCherry+ cells/mm^2^ (mean +/− SD, *n* = 3 retinas) are visible with our imaging conditions. 

In vivo imaging of RGC disease modeling has the potential to transform mouse RGC disease model research. The specificity of mCherry expression combined with its long-term presence in RGCs makes this reporter mouse an excellent tool for studying RGC disease models and treatment interventions. 

## 3. Conclusions

Currently, standard methods to visualize RGCs require sacrificing experimental animals for staining/labeling or are not RGC-specific (e.g., conventional OCT imaging or Thy1-YFP imaging using SLO). Therefore, they are not ideal for monitoring RGCs in living animals and to assess glaucomatous disease progression in real-time. In this report, we have developed a new mouse strain in which all Brn3b-expressing cells express the mCherry fluorescent protein without interfering with normal Brn3b function. In the retina of both adult and developing Brn3b-mCherry mice, mCherry is specifically expressed in a large proportion of RGCs. Furthermore, the level of mCherry expression is sufficient for us to detect RGCs using non-invasive methods in live animals (SLO). Therefore, this new mouse line can be used for developmental studies and, if combined with existing glaucoma models, will enable longitudinal monitoring of RGCs using the fluorescent channel of SLO. Interestingly, a widespread decrease in gene expression, including Brn3, has been reported in glaucoma models [80,81]. This decline in Brn3 and other genes correlates with retrograde axonal transport deficiencies and degeneration. Therefore, Brn3b levels and consequently mCherry will not necessarily reflect cell loss in experimental models of glaucoma but it is a convenient readout of the early stages of RGC degeneration. We believe this tool will greatly facilitate the validation of new therapies and neuroprotection strategies for glaucomatous degenerations.

## 4. Materials and Methods

### 4.1. Animals

All mice husbandry and handling were in accordance with protocols approved by the University of California Davis Animal Care and Use Committee (IACUC protocol # 19413, approved on 12July2016), which strictly adheres to all NIH guidelines and satisfies the Association for Research in Vision and Ophthalmology guidelines for animal use. The Brn3b-mCherry CRISPR knock-in mouse line was generated by Biocytogen (Worcester, MA, USA) in the C57BL/6N background. We have not performed whole-genome sequencing to screen for off-target effects but we have not detected any abnormalities, viability, fertility or any developmental problems (4 different generations have been analyzed to date). For all the embryonic analyses, the morning of the vaginal plug was considered E0.5. The animals are currently being bred to a different background to avoid the Rd8 mutation present in the C57BL/6N background. We will make this line available upon request to the scientific research community.

### 4.2. Immunohistochemistry

For cryosection, embryonic whole heads and P0 dissected eyes were collected and fixed for 30 min at 4 °C in 4% Paraformaldehyde (PFA, Cat # 15714, Electron Microscopy Sciences for 32% stock, Hatfield, PA, USA). Following fixation, tissues were cryopreserved by sequential gradients of 10%, 20%, 30% sucrose (tissues were maintained in each solution until they sink), and finally a mixture of half 30% sucrose and half O.C.T. (Tissue-Tek^®^ O.C.T. Compound, Sakura^®^ Finetek, Alphen aan den Rijn, Netherlands. Cat # 4583). Tissues were finally embedded in O.C.T., quickly frozen using dry ice, and stored at −80 °C until sectioning. For paraffin samples, eyes were collected and frozen in dry-ice chilled propane, and freeze-substituted with methanol-acetic acid at −80 °C, as described previously [82], transferred to ethanol and embedded in paraffin. Paraffin sections were deparaffinized using Xylene, and further rehydrated. After de-paraffinizing, tissues were washed with PBS, and antigen retrieval was performed with Sodium Citrate Buffer (10mM Sodium Citrate, 0.05% Tween20, pH 6.0). Briefly, the slides were placed in a microwave-safe vessel in the Sodium Citrate Buffer and the microwave was set to full power until the solution boiled. The samples were allowed to cool down and were then rinsed 5 times with PBS. Subsequently, all tissue was blocked in 10% Normal Donkey Serum (NDS) in Phosphate Buffered Saline (PBS)-0.1% Triton X-100, and the samples were incubated with primary antibodies diluted in fresh blocking solution overnight at 4°C. Primary antibodies used include: Goat anti-Brn3 (Santa Cruz, Dallas, TX, USA, #SC-6026, 1:100), Mouse anti-Tuj1 (Biolegend, San Diego, CA, USA, #801201, 1:500), Rabbit anti-mCherry (Novus Biologicals, Centennial, CO, USA, #NBP2-25157, 1:500), Goat anti-mCherry (Acris Antibodies, Rockville, MD, USA. #AB0040-200, 1:500), Guinea Pig anti-RBPMS (Phosphosolutions, Aurora, CO, USA, #1832-RBPMS, 1:500), Rabbit anti-Sncg (generous gift from Nick Marsh-Arsmtrong, Princeton, NJ, USA, 1:10,000), Rabbit anti-Atoh7 (Novus Biologicals, #88639, 1:200), anti-Pax2 (Biolegend #901001, 1:1000), anti-PCNA (Invitrogen, Rockford, IL, USA, #13-3900, 1:100). Next, tissues were rinsed 5 times with PBS and incubated with Alexa Fluor secondary antibodies (Thermo Fisher Scientific, Rockford, IL, USA) in blocking solution. All tissues were counterstained with 4′,6-diamidino-2-phenylindole (DAPI). The sections were rinsed with PBS-0.1% Triton and mounted for microscopy with Fluoromount-G (Southern Biotech, Birmingham, AL, USA). Most images were obtained with a 20× oil objective and captured with an Olympus FV1000 confocal microscope. High-magnification images were obtained with a 40× oil objective. Images were assembled in Adobe Photoshop and Illustrator. Brightness and contrast were similarly adjusted to all samples. 

### 4.3. Retinal Ganglion Cell Counts

Adult retinas were flat-mounted and stained for mCherry and RBPMS (*n* = 4 mice from two different generations). RBPMS labels all RGCs and thus was used as the denominator to count the total number of RGCs in the retina. The percentage of RGCs labeled was measured as the total number of mCherry positive cells over RBPMS positive cells. For each retina, at least 3 pictures from different regions of the eye (ventral, dorsal and central) were taken, quantified and averaged. To calculate mCherry+ cell size, digital images of 250 × 250 μm^2^ of flat-mounted retinas were processed to set intensity and background levels, and mCherry+ cell diameters were calculated using Fiji, considering diameter as the longest distance between any two points on a cell’s perimeter.

### 4.4. In-Vivo Retina Cellular Imaging

For in vivo retinal imaging experiments, mice were anesthetized with the inhalational anesthetic isoflurane (2–3% in O_2_), and their pupils were dilated with medical grade tropicamide and phenylephrine. A contact lens and gel (GenTeal Tears, Alcon, Fort Worth, TX, USA) was used to maintain the cornea transparency during in vivo retinal imaging [83]. Mouse body temperature was maintained with a temperature-controlled blanket under the animal to prevent cooling of mouse body and development of a cold-cataract. The mouse head was stabilized by a customized bite-bar connected with positioning stage.

A custom rodent scanning laser ophthalmoscopy (SLO) detection channel of custom multimodal mouse retinal imaging instrument [79] was used to image back reflected and fluorescence signal from the mouse retina, with excitation light from an OBIS LX 561nm laser (Coherent Inc., U.S.), and a long-pass filter (BLP02-561R, Semrock, Rochester, New York, USA) to select the emission light for mCherry-expressing cells. The power at the mouse pupil was ~300 μW, and the beam diameter at the mouse pupil was ~0.5mm which offers a lateral resolution of 2.9 μm [84]. 

The mouse retina was first imaged with full field-of-view (FOV) of 51 degrees, corresponding to ~2 mm on the mouse retina, to allow search for the region of interested (ROI). Then a 3× zoom-in region was selected and imaged with higher density sampling. For each ROI, a total of 100 serial SLO images, including both reflectance and fluorescence, were collected. The serial images were further registered to using ImageJ TurboReg plugin with ‘Rigidbody’ transformation [85], and then averaged for display. 

## Supplementary Materials

Supplementary materials can be found at https://www.mdpi.com/1422-0067/20/12/2903/s1.
