# Supplementary material for: A Novel Reporter Mouse Uncovers Endogenous Brn3b Expression
Source: Int J Mol Sci. 2019 Jun 14;20(12):2903. doi: 10.3390/ijms20122903 (PMC6627301; doi:10.3390/ijms20122903)
Supplement: Supplementary file 1 [file ijms-20-02903-s001.zip › Suppl Table1.pdf]

Brn3b-mCh retinas	Average	stdev	C57BL/6J retinas	Average	stdev
**mCh+/mm2**	2297	412	**mCh+/mm2**	na	na
**Brn3+/mm2**	2319	413	**Brn3+/mm2**	2063	262
**RBPMS+/mm2**	3244	538	**RBPMS+/mm2**	2941	824
**mCh/RBPMS (%)**	70.86	4.33	**mCh/RBPMS (%)**	na	na
**Brn3/RBPMS (%)**	71.73	2.73	**Brn3/RBPMS (%)**	71.95	11.96
**mCh/Brn3 (%)**	99.06	2.56	**mCh/Brn3 (%)**	na	na
