# Supplementary figures and images for: A Novel Reporter Mouse Uncovers Endogenous Brn3b Expression
Source: Int J Mol Sci. 2019 Jun 14;20(12):2903. doi: 10.3390/ijms20122903 (PMC6627301; doi:10.3390/ijms20122903)

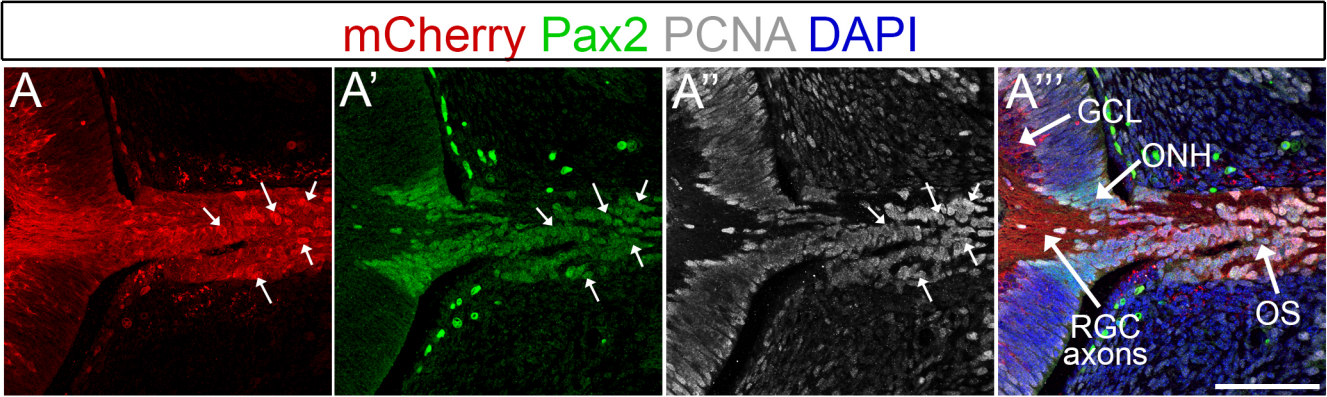

Supplement: Supplementary file 1 [file ijms-20-02903-s001.zip › S2.pdf]

A

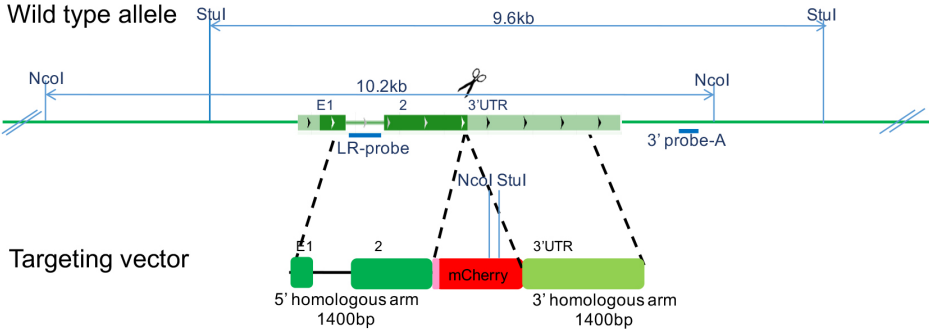

B

| Restriction enzyme | Probe   | WT     | Targeted |
|--------------------|---------|--------|----------|
| NcoI               | LR (5') | 10.2kb | 8.3kb    |
| Stul               | 3'      | 9.6kb  | 6.5kb    |

Supplement: Supplementary file 1 [file ijms-20-02903-s001.zip › S1.pdf]
