# Supplementary material for: A Novel Reporter Mouse Uncovers Endogenous Brn3b Expression
Source: Int J Mol Sci. 2019 Jun 14;20(12):2903. doi: 10.3390/ijms20122903 (PMC6627301; doi:10.3390/ijms20122903)
Supplement: Supplementary file 1 [file ijms-20-02903-s001.zip › Supplementary figure legends_REBUTTAL.docx]

**Supplementary Figure 1:**

Southern blot strategy design.

**Supplementary Figure 2:**

**mCherry expression in the optic stalk.** Co-localization experiments with mCherry (red), Pax2 (green), PCNA (gray) and counter-stained with DAPI (blue). White arrows in A-A’’ indicate mCherry+ Pax2+ PCNA+ cells present in the optic stalk. GCL: ganglion cell layer, ONH: optic nerve head, RGC: retinal ganglion cells, OS: optic stalk. Scale bar: 100 microns.

**Supplementary Table 1:** Cell counts of mCherry-Brn3b (n=4) and wild type C57/BL6J (n=3) retinas. na: not applicable.
